# Microstructure and Compression Properties of V_SS_-V_3_B_2_ Eutectic Alloys in the V-Si-B System

**DOI:** 10.3390/ma13092100

**Published:** 2020-05-01

**Authors:** Christopher Müller, Georg Hasemann, Maximilian Regenberg, Ulf Betke, Manja Krüger

**Affiliations:** Institute of Materials and Joining Technology, Otto-von-Guericke University Magdeburg, Universitätsplatz 2, 39106 Magdeburg, Germany; georg1.hasemann@ovgu.de (G.H.); maximilian.regenberg@ovgu.de (M.R.); ulf.betke@ovgu.de (U.B.); manja.krueger@ovgu.de (M.K.)

**Keywords:** V-Si-B, V-B, vanadium-based alloys, intermetallics, microstructure characterization, mechanical properties, compression test, experimental data

## Abstract

The present study reports on the microstructural evolution and room temperature plasticity of V(-Si)-B alloys with respect to the V solid solution (V_SS_)-V_3_B_2_ phase region. To investigate the occurring effects systematically, different binary V-B and ternary V-Si-B alloys were produced by conventional arc melting. Scanning electron microscope (SEM) analyses and X-ray diffraction (XRD) measurements were used to characterize the resulting as-cast microstructures. For the first time, the eutectic composition was systematically traced from the binary V-B domain to the ternary V-Si-B system. The observations discover that the binary eutectic trough (V_SS_-V_3_B_2_) seems to reach into the ternary system up to an alloy composition of V-5Si-9B. Room temperature compression tests were carried out in order to study the impact of single-phase and multi-phase microstructures on the strength and plasticity of binary and ternary alloys. The results indicate that the V_SS_ phase controls the plastic deformability in the V_SS_-V_3_B_2_ eutectic microstructure whereas the intermetallic V_3_B_2_ acts as a strong hardening phase.

## 1. Introduction

V-Si-B alloys provide great potential as a new type of structural lightweight material for high temperature applications, e.g., in turbines for energy conversion, due to the high melting point of vanadium, T_m_ = 1910 °C [1], and a comparable stress-strain behavior as the state-of-the-art Ni-based superalloy CMSX-4 at 1100 °C in combination with a low density of ρ = 5.21–6.11 g/cm^3^ [2,3,4]. Thus, to replace Ni-based superalloys, they are in competition with Nb-, Mo- or Pt-based high temperature materials that have been the focus of research for several years [5,6,7,8,9].

A disadvantage of vanadium compared to the other base metals, as mentioned above, is its high affinity for oxygen and the associated high tendency to oxidize. Investigations on vanadium alloys have shown that the alloying elements Si and B can reduce the formation of vanadium oxides and, therefore, positively influence the oxidation properties at high temperatures [10,11]. The formation of protective SiO_2_ scales and the emergence of low-viscosity borosilicate glass by adding B make the ternary V-Si-B system appear promising for potential use in high temperature applications [10,11,12,13,14]. However, low-alloyed V materials will need an environmental barrier coating if used at temperatures higher than 500 °C.

**Figure 1:**
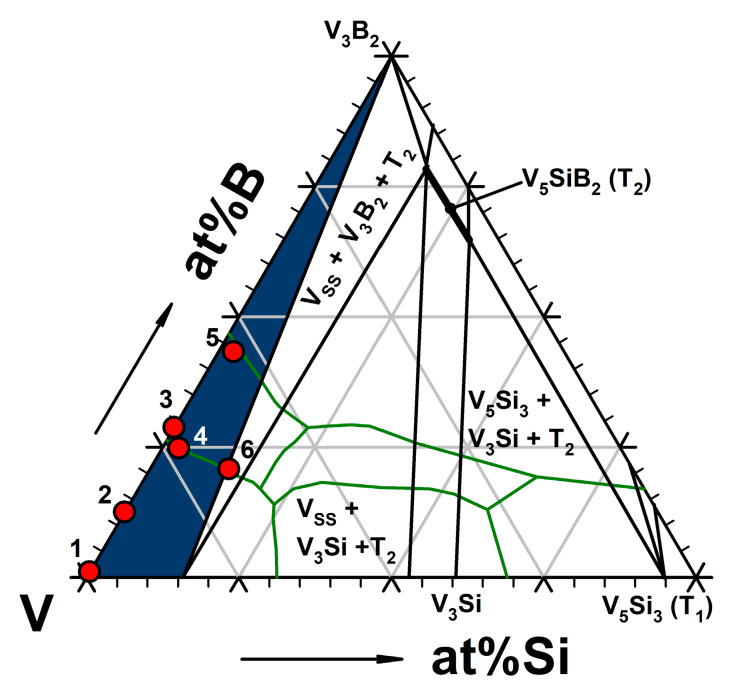
Isothermal section at 1600 °C of the V-Si-B system after Nunes [17] including investigated alloys (red spots, compositions are given in Table 1) in the V_SS_-V_3_B_2_ phase region (blue area) and the liquidus projection [19] of the V-rich corner (green line).

The first research activities on the V-Si-B system were performed by Nowotny and Kudielka, who published an isothermal section of the V-Si-B system at 1450 °C [15,16]. Together with the efforts of Nunes et al. [17], who published an isothermal section of the V-rich corner at 1600 °C (Figure 1), these first examinations provided the foundation for current microstructural investigations in the ternary V-Si-B system. It was also found that the solubility of boron in the vanadium solid solution phase (V_SS_) and in the silicide phases (V_3_Si, V_5_Si_3_, V_6_Si_5_), as well as that of Si in boride phases (VB and V_3_B_2_), is negligible [17]. Da Silva et al. [18] published a calculated and in parts experimentally evaluated liquidus projection of the V-Si-B system, which summarizes the previous efforts regarding the microstructural evolutions in the ternary system. For the V-rich corner, he presented three different ternary eutectic reactions: L ↔ V_SS_ + V_3_B_2_ + V_5_SiB_2_ (1729 °C), L ↔ V_3_Si + V_SS_ + V_5_SiB_2_ (1741 °C) and L ↔ V_5_Si_3_ + V_3_Si + V_5_SiB_2_ (1848 °C). Hasemann [19] recently published a study of the liquidus surface in the V-rich corner of the V-Si-B system (as highlighted green in Figure 1). He reported on the solidification behavior of more than 40 different arc-melted alloys. The findings of Hasemann in part confirm the calculations of da Silva et al. [18], but also lead to new information about the invariant reactions. In contrast to the work of da Silva, only two ternary eutectic reactions could be experimentally confirmed and no evidence for the eutectic reaction L ↔ V_SS_ + V_3_B_2_ + V_5_SiB_2_ was found. Hasemann [19] rather reported on a peritectic transformation L + V_3_B_2_ ↔ V_SS_ + V_5_SiB_2_ that was not described before. The previous investigations of the V-Si-B system [15,16,17,18,19] presented important information regarding solubility ranges, primary solidifying phases and invariant reactions in the V-Si-B system. However, the influence of the oxygen content (V-Si-B-O) on phase equilibria, solid solubility and reaction compositions was not explicitly considered in any of the publications mentioned. It cannot be excluded that deviations have arisen as a result.

V-Si-B alloys show competitive mechanical properties at 1000 °C as compared to the well-known nickel-based superalloy CMSX-4 [14]. Recent studies have also shown that multi-phase V-Si-B alloys, unlike the similarly composed Mo-Si-B alloys, exhibit plasticity at room temperature. This seems to be due to the V_SS_ phase [20,21]. Henshall et al. [20] showed that the volume fraction of the V_SS_ phase can be used to control the fracture toughness of binary V_SS_-V_3_Si alloys. Hasemann et al. [21] recently showed that the volume fraction of the V_SS_ phase completely controls the plastic deformability in ternary V-Si-B alloys, consisting of the V_SS_ phase (A2 structure), V_3_Si (A15 structure) and V_5_SiB_2_ - the latter is the so-called T_2_ phase (D8_l_ structure). Dislocations could only be observed in the solid solution phase, which has the largest volume fraction in the ternary eutectic V_SS_-V_5_SiB_2_-V_3_Si [21,22]. It is noticeable that the information on the V_SS_-V_3_B_2_ subsystem [23,24] in the ternary V-Si-B system is quite limited in contrast to the V_SS_-V_3_Si subsystem [19,20,21,22,25]. Therefore, the present study focuses on the V_SS_-V_3_B_2_ phase region (as highlighted blue in Figure 1), the emerging microstructures of selected alloys, eutectic reactions and the resulting mechanical properties.

Spear et al. [23] assessed the binary V-B phase diagram, which later was extended and corrected by de Lima et al. [24]. They reinvestigated the compositions close to the invariant reactions in the V-B system. For the eutectic reaction L ↔ V_SS_ + V_3_B_2_, a concentration of 12 at.% B was experimentally determined and evidence of a peritectic reaction (L + VB ↔ V_3_B_2_) was already detected at 19 at.% B instead of 25 at.% B, as was previously assumed. Da Silva et al. [18] calculated a boron concentration of 11.3 at.% B (1732 °C) for the eutectic reaction and a slightly lower B-concentration of 18.7 at.% B (1905 °C) for the peritectic reaction. These calculations are in good agreement with the experimental results of de Lima et al. [24]. The alloy compositions in this work were carefully chosen based on these experimental and calculated results. With respect to the mechanical properties of V_SS_-V_3_B_2_ eutectic and near-eutectic alloys, presently no information is available in the literature.

The present study aims to investigate the alloying effects of B on the microstructure formation, second phase hardening and room temperature mechanical properties of near-eutectic V_SS_-V_3_B_2_ alloys. Therefore, different binary V-B and ternary V-Si-B alloys were chosen. To investigate the influence of primary solidification and the volume fraction of the participating phases, alloys in different primary solidification areas, V_SS_ and V_3_B_2_ (D5_a_ structure) [18,23], were selected.

## 2. Materials and Methods

All alloys were produced by conventional arc melting under argon atmosphere in a water-cooled copper crucible. V (min. 99.7%), Si (min. 99.99%), and B (min. 99.0%) flakes were used as starting materials. In order to achieve homogeneous samples, the buttons were flipped and re-melted five times. Each button had a weight of 15 g and a diameter between 20 and 25 mm. The measured mass change after melting was negligible for all samples (< 1 wt.%). All investigations within the present study were performed in the as-cast conditions. The actual alloy compositions were examined by inductively coupled plasma optical emission spectroscopy (ICP-OES, measuring accuracy < 3%) by dissolving sample pieces in HCl + HNO_3_ + HF and using a Thermo Scientific iCAP6500 spectrometer. The concentration of oxygen (in wt.ppm) was determined in accordance with the carrier gas procedure via melt extraction with subsequent infrared detection using a Bruker G8 Galileo System. The results of chemical analyses and microstructural characteristics of the alloys (primary phase, volume fraction of phases) are summarized in Table 1.

In order to study the microstructure of the as-cast alloys, they were cut by means of electrical discharge machining (EDM). The hot mounted (Struers Poly Fast) samples were ground from 180 grit down to 2000 grit, followed by mechanical polishing with a 3 and 1 μm diamond suspension and finished using colloidal silica. The microstructures were investigated using a Zeiss Merlin or Zeiss Supra 50 VP scanning electron microscope (SEM). SEM images were typically performed in the backscattered electron (BSE) mode. In addition, electron backscatter diffraction (EBSD) measurements (Oxford Instruments) of deformed samples were used to visualize the local changes in orientation within the crystal structure as an indicator of plastic deformation. Standardless energy dispersive X-ray spectroscopy (EDS, Oxford Instruments) was used to assign phases (identified using X-ray diffraction (XRD) measurements) during SEM analyses and to check for the presence of Si in the V_3_B_2_ phase.

**Figure 2:**
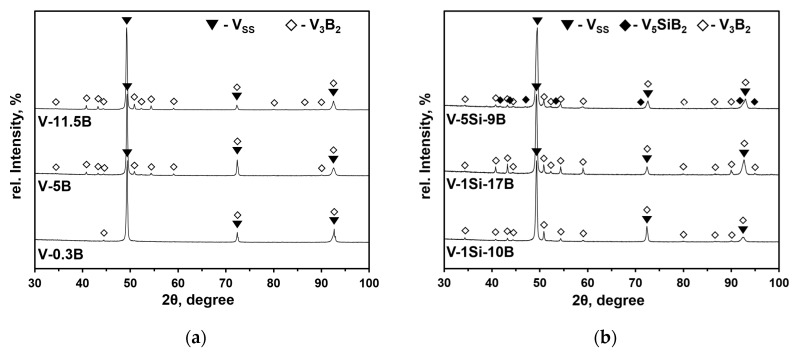
X-ray diffraction (XRD) pattern of (**a**) V-B alloys; (**b**) V-Si-B alloys.

The identification of phases present in the V(-Si)-B alloys were determined by X-ray diffraction (XRD) in a θ/θ reflection geometry with a 2θ range from 20° to 160° using a PANalytical X’Pert Pro Bragg-Brentano diffractometer and Co-Kα_1_/α_2_ radiation. The obtained diffraction patterns are summarized in Figure 2. They were analyzed by the Rietveld technique using the Topas Academic 5 program package [26] to determine the weight fraction of the phases together with their crystallographic density. From these data, the volume fractions of the respective components were calculated. The crystallographic data of V and V_3_B_2_ necessary for the Rietveld analyses were obtained from the “Crystallography Open Database” (COD, deposition numbers: 1510850 for V_3_B_2_ [27] and 9012770 for V [28]). The structural data of the V_5_SiB_2_ phase were adopted from the isotypic Mo_5_SiB_2_ compound, which was structurally characterized by neutron diffraction in 2001 [29]. The adoption of the Mo_5_SiB_2_ type for V_5_SiB_2_ was recently confirmed by first principles calculations [30]. For each phase, a parameter set was refined consisting of the lattice parameters, a scale parameter correlated to the weight fraction and profile parameters for approximating the reflection profile. For the V_SS_, a significant amount of preferred orientation of the crystallites was observed, resulting in deviations in the reflection intensities. This was accounted for by including a spherical harmonics function of the 8th order as implemented in Topas Academic [31]. The non-special atomic coordinates were refined for the V atom located on site 16*l* in V_5_SiB_2_ and the V atom on site 4*h* in V_3_B_2_, all other atomic coordinates were either special sites or left on their initial values. Global parameters refined was a 15th order Chebychev polynomial for background modelling. 

Compression tests were performed in order to investigate the mechanical properties of the alloys. The compression tests were executed at room temperature using an Instron 1381 and a Zwick/Roell Z100 electro-mechanical universal testing machine. Compression samples with 2.5 mm length and a diameter of 1.5 mm were prepared by EDM. The tests were performed at an initial (engineering) strain rate of $\dot{\varepsilon }$ = 10^−3^s^−1^. The yield stresses were determined by the 0.2% offset method.

## 3. Results and Discussion

### 3.1. Binary V_SS_-V_3_B_2_ Alloys

**Figure 3:**
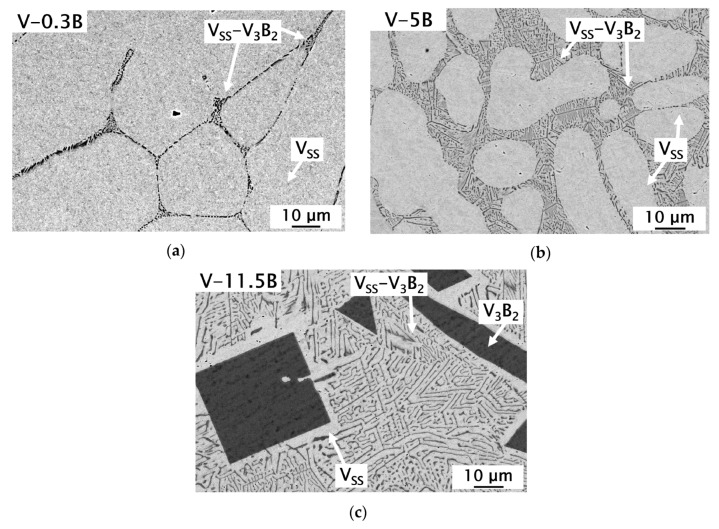
Scanning electron microscopic images in the backscattered electron mode (SEM-BSE), showing the as-cast microstructure of the binary V-B alloys: (**a**) V-0.3B; (**b**) V-5B; (**c**) V-11.5B with small V_SS_ halos around V_3_B_2_ primary phase.

**Figure 4:**
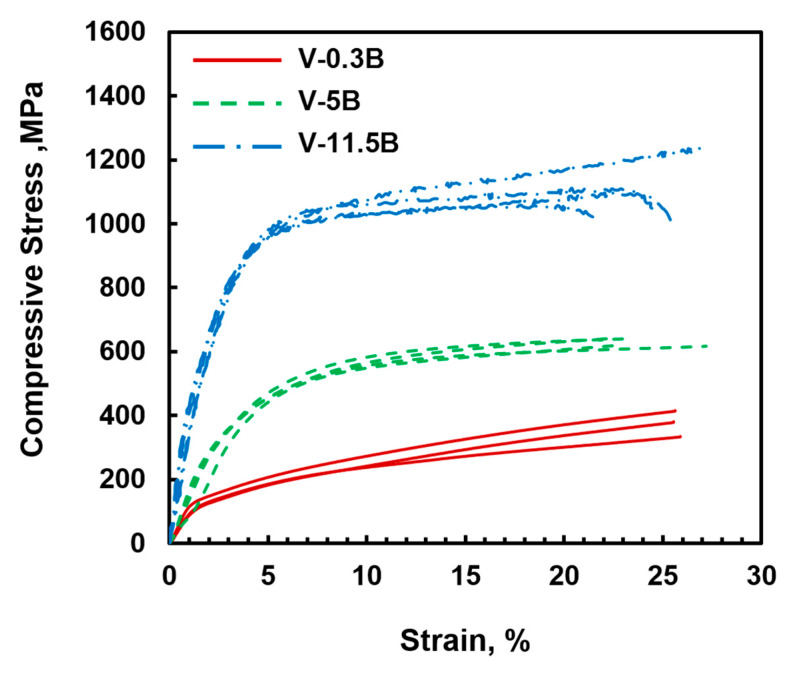
Compressive engineering stress vs. strain curves at room temperature of alloys V-0.3B, V-5B and V-11.5B.

**Figure 5:**
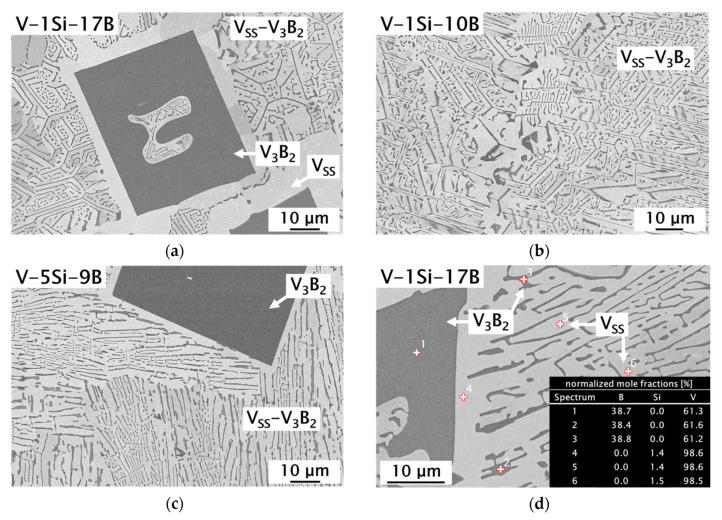
SEM-BSE images showing the as-cast microstructure (in part with small V_SS_ halos around the primary V_3_B_2_) of the ternary V-Si-B alloys: (**a**) V-1Si-17B; (**b**) V-1Si-10B; (**c**) V-5Si-9B and (**d**) results of EDS spot analyses for V-1Si-17B.

Three different alloy compositions were used to investigate the phase formation in binary V-B alloys with respect to the binary eutectic composition (L ↔ V_SS_ + V_3_B_2_) and their mechanical properties at room temperature with an increasing volume fraction of V_3_B_2_. The as-cast microstructures of the alloys are shown in Figure 3. All three alloys are located in the two-phase region consisting of the V_SS_ and the V_3_B_2_ phase. V-0.3B (Figure 3a) and V-5B (Figure 3b) alloys solidify within the V_SS_ primary crystallization field, while the V-11.5B (Figure 3c) solidifies primarily with V_3_B_2_.

At the grain boundaries of the V-0.3B alloy (Figure 3a), small eutectic V_SS_-V_3_B_2_ areas were formed. This indicates that the solubility of B in V is well below 0.3 at.% B and may even further decrease if equilibrium conditions are considered. It has to be mentioned that longer polishing times were necessary for the alloy V-0.3B and therefore etching effects occurred in the V_SS_. The hypoeutectic alloy V-5B consists of large V_SS_ dendrites surrounded by the binary V_SS_-V_3_B_2_ eutectic. The volume fraction of V_SS_ phase is visibly decreased in contrast to V-0.3B (see Table 1). The alloy V-11.5B, Figure 3c, shows a minor volume fraction of the primary V_3_B_2_ phase surrounded by the non-facetted–facetted V_SS_-V_3_B_2_ eutectic microstructure. In the absence of quantitative wavelength-dispersive X-ray spectroscopy (WDS) data about the chemical composition of the eutectic, it is suggested that the chemical composition of this alloy as determined by ICP-OES can be considered as the eutectic composition. V_SS_ halos can be observed around the primary V_3_B_2_ phase in alloy V-11.5B as it was already documented in [24]. The present experimental findings are in good agreement with previous work by de Lima et al. [24], Hasemann [19] and the thermodynamic calculations by da Silva et al. [18].

The non-facetted (V_SS_)–facetted (V_3_B_2_) eutectic structure as well as the formation of V_SS_ halos surrounding the primary V_3_B_2_ phase in alloy V-11.5B can be explained with the difference in melting entropy ΔS_m_ of either the V_SS_ and V_3_B_2_ phase. In order to classify eutectic structures, ΔS_m_ or the so-called α-factor, Equation (1), can be used [32,33], where R_g_ is the universal gas constant:

α = ΔS_m_ / R_g_ (1)

Sahm et al. [32] described the facetted formation for phases when α > 2 and the non-facetted formation for phases if α < 2 within eutectic structures. Using the appropriate ΔS_m_ for V_SS_ and V_3_B_2_ (calculated using CALPHAD method [34] and ThermoCalc, version 2015b, database TCBIN 1.1), the α-factor according to Equation (1) is α(V_SS_) = 1.2 and α(V_3_B_2_) = 2.1. Therefore, it can be assumed that the V_SS_ phase grows non-facetted and V_3_B_2_ grows facetted within the V_SS_-V_3_B_2_ eutectic microstructure.

The halo formation surrounding the primary V_3_B_2_ phase can also be explained by the difference in ΔS_m_ between V_SS_ and V_3_B_2_ [32,33]. The difference of ΔS_m_ between both eutectic phases results in an asymmetrical coupled zone of eutectic growth, which results in a faster growth of the eutectic as compared to the growth of the individual primary phase. Thus, the coupled zone is shifted towards the phase with the higher ΔS_m_ (here V_3_B_2_) value. Consequently, and according to Kurz and Sahm [33], a primary phase formation of V_3_B_2_ becomes possible at the eutectic composition. Subsequent to the V_3_B_2_ nucleation, the chemical composition of the liquid phase changes following the liquidus line of V_3_B_2_ and their extension beneath the eutectic temperature as a metastable liquidus line, until the nucleation of V_SS_ starts. If the composition of the remaining liquid phase is then located within the coupled zone, the eutectic growth of V_SS_-V_3_B_2_ starts directly at the V_SS_ phase that has formed the halo [32,33].

In the engineering stress vs. strain diagram in Figure 4, the results of the room temperature compression tests for the binary V-B alloys are shown. Three to five samples were tested for each alloy, showing a good reproducibility of the compressive stress-strain behavior. All investigated V_SS_-V_3_B_2_ materials show high deformability at room temperature, which is in good agreement with the recently published results for different V-Si-B alloys [21]. Despite the decrease in the volume fraction of V_SS_ phase (Table 1), no drop in deformability can be observed between V-0.3B and V-11.5B. In contrast, as the B content increases, a significant increase in strength can be observed. The yield stress increases from 100 ± 20 MPa for 0.3 at.% B to 270 ± 40 MPa for 5 at.% B and 450 ± 40 MPa for the alloy V-11.5B. As already mentioned, the solubility of B in the V_SS_ phase is negligible [23] and therefore does not contribute significantly to the hardening of the alloys. It can therefore be assumed that the presence of the V_3_B_2_ phase as well as the fine eutectic microstructure are responsible for the observed increase in strength. With respect to recent work [21], it is noticeable that silicon seems to have a higher impact on the solid solution hardening in vanadium than boron.

### 3.2. V_SS_-V_3_B_2_ Alloys with Si Additions

In this work, special attention was paid to the microstructure evolution and mechanical properties as a function of the boron and silicon concentration in V(-Si)-B alloys. Two near eutectic V-Si-B alloys (V-1Si-10B and V-5Si-9B) were prepared to follow the eutectic line through the corresponding V_SS_-V_3_B_2_ two-phase region [18,19] and one hypereutectic alloy (V-1Si-17B) to investigate second phase strengthening due to higher V_3_B_2_ contents. The influence of Si on the solid solution and the second phase strengthening can be studied, since Si acts as a strong solid solution strengthener and can be solved in the V_SS_ phase up to comparatively high concentrations of around 7 at.% [1,21].

Figure 5 shows the resulting as-cast microstructures of the examined V-Si-B alloys. As demonstrated in Figure 5a, V_3_B_2_ is the primary phase in alloy V-1Si-17B. Due to the hypereutectic alloy concentration, a significant increase in the volume fraction of V_3_B_2_ and a concomitant decrease in the V_SS_ phase are discernible for V-1Si-17B compared to the binary alloys. In the peripheral areas around the borides, a V_SS_ halo has formed, delimiting the borides from the surrounding V_SS_-V_3_B_2_ matrix. Like the binary alloy V-11.5B, V-1Si-10B (Figure 5b) solidifies in a eutectic microstructure with fine binary eutectic V_SS_-V_3_B_2_. Nunes et al. [17] determined the solid solubility of Si in V_3_B_2_ by wave length-dispersive X-ray spectroscopy (WDS) to be negligible, which agrees well with present EDS analyses illustrated in Figure 5d (as an example V-1Si-17B, spot 1-3). For all alloys examined, the presence of silicon could not be detected in V_3_B_2_. Therefore, it can be concluded that silicon is completely dissolved in the V_SS_ phase, which is in agreement with the WDS analyses in our previous work [21]. In addition, it can be deduced from the microstructure investigations that alloying with 1 at.% Si slightly shifts the V_SS_-V_3_B_2_ eutectic trough in V-Si-B alloys towards lower B contents. Due to its composition, the alloy V-5Si-9B follows the binary eutectic trough into the ternary V-Si-B system. The corresponding microstructure is shown in Figure 5c. Under as-cast conditions, a eutectic microstructure with only minor portions of the primary V_3_B_2_ phase emerges. EBSD analyses show that the eutectic regions consist of V_SS_, V_3_B_2_ and small amounts of V_5_SiB_2_ (Figure 7a), which is in good agreement with the XRD analysis. EBSD is needed since both intermetallic phases show electron channeling using BSE images and are therefore hard to distinguish [21,22]. Due to these findings, the alloy V-5Si-9B is considered to be located in the V_SS_-V_3_B_2_-V_5_SiB_2_ three-phase region [17]. In contrast to the calculated liquidus projection by da Silva et al. [18], it can be assumed that the V_SS_-V_3_B_2_ eutectic valley is extended towards higher Si concentration. This observation is consistent with the findings of Hasemann [19].

**Figure 6:**
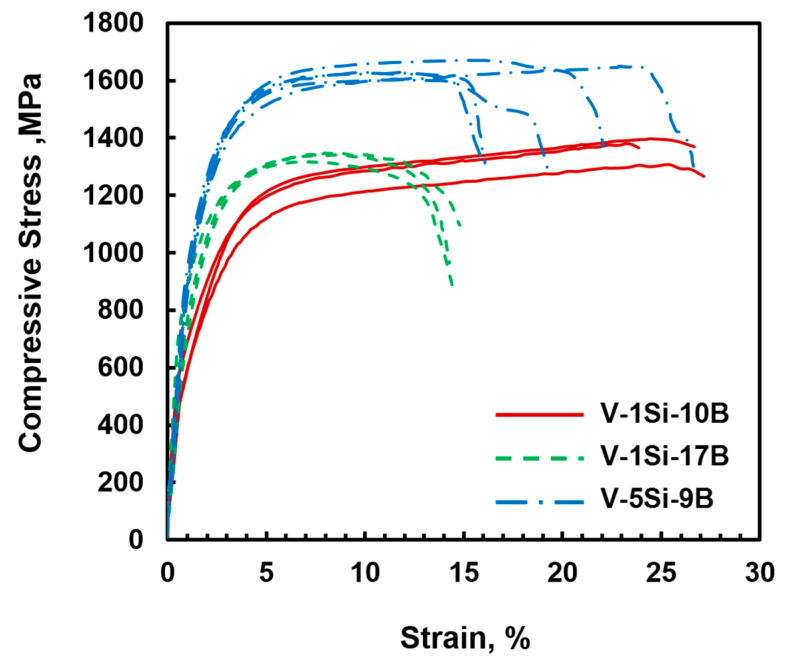
Compressive engineering stress vs. strain curves at room temperature of alloys V-1Si-10B, V-1Si-17B and V-5Si-9B.

In order to estimate the mechanical properties of the ternary V-Si-B alloys as well as the impact of Si on V_SS_-V_3_B_2_ alloys, room temperature compression tests were performed. Figure 6 illustrates the resulting compressive engineering stress vs. strain curves. In the case of V-1Si-10B, the compressive yield stress of 670 ± 100 MPa is higher than for V-11.5B. Both alloys show the same plastic deformability; however, the silicon addition in alloy V-1Si-10B causes additional solid solution hardening. Thus, an increase in the yield stress of about 200 MPa relative to V-11.5B can be observed and is in good agreement with recent observations [21]. With an average compressive yield stress of 820 ± 25 MPa for the alloy V-1Si-17B, there is a noticeable increase in strength compared to the alloy V-1Si-10B. Since the Si content was kept constant, it can be assumed that the increase can be attributed to a higher boron content and a higher V_3_B_2_ volume fraction resulting from the large primary V_3_B_2_ phases. In addition, a significant reduction in deformability at room temperature can be observed. For the alloy V-5Si-9B, the mechanical properties result in a compressive yield stress of around 990 ± 50 MPa. In contrast to V-1Si-10B, the stress values can be explained due to an increased Si content and thereby increased solid solution strengthening. In combination with the almost constant volume fraction of the V_SS_ phase (~80 vol.%), the deformability at room temperature also remains exceptionally high, with at least 14% of total compressive strain. It cannot be excluded that mechanical properties in the present work were influenced by the oxygen content of the alloys (V_SS_ + O, Si + O). However, the compressive strains measured do not indicate significant embrittlement.

**Figure 7:**
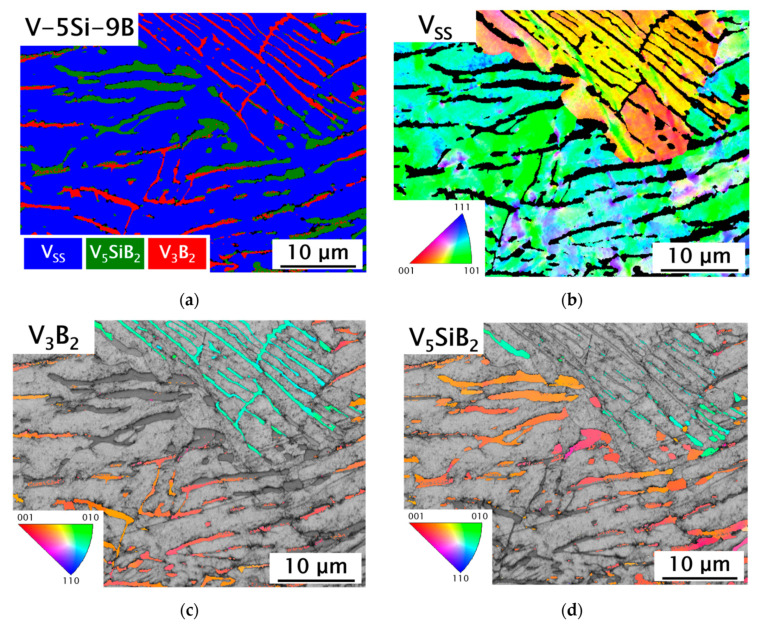
**Electron backscatter diffraction** (EBSD) phase and orientation maps of deformed V-5Si-9B (15kV, step size = 0.088 μm): (**a**) phase map of V-5Si-9B; orientation maps (inverse pole figure colored, in sample normal direction) for (**b**) V_SS_ (vanadium solid solution phase); (**c**) V_3_B_2_; (**d**) V_5_SiB_2_.

The results of EBSD observations on deformed samples prove that plasticity at room temperature in V_SS_-V_3_B_2_ alloys is exclusively controlled by the V_SS_ phase. Figure 7 shows an EBSD phase map and orientation maps for the deformed alloy V-5Si-9B (after compression test). The local crystal orientation changes within the V_SS_ areas (Figure 7b) of a single grain due to dislocation movement and thus, plastic deformation. The orientation maps of the intermetallic phases V_3_B_2_ and V_5_SiB_2_ (Figure 7c,d) did not result in any significant differences of orientation within single grains, which assumes no dislocation activities at room temperature.

In combination with recent investigation on the room temperature deformability of V-Si-B alloys [21], it is possible to estimate the individual strength contributions in the ternary alloys. In the work of Hasemann et al. [21], compression yield stresses for the alloys V-1Si and V-5Si were measured. Since the samples were produced and tested in the same way, the direct comparison with the yield stresses measured in this work can be drawn. Therefore, Si contributes by about 300 MPa to the strength of the alloys V-1Si-10B and V-1Si-17B. For the alloy V-5Si-9B, the solid solution hardening contribution of Si can be estimated to approx. 600 MPa [21]. The corresponding shares of the yield stresses, which result from the amount of V_3_B_2_ and V_5_SiB_2_ in the microstructure, can therefore be estimated within the near-eutectic alloys V-1Si-10B and V-5Si-9B with about 400 MPa. For the hypereutectic alloy V-1Si-17B, the increased volume fraction of second phase V_3_B_2_ (41.5 vol.%) contributes approx. 500 MPa to the measured yield stress.

## 4. Summary and Conclusions

The present study reports on the microstructural evolution of as-cast V_SS_-V_3_B_2_ eutectic alloys in the V(-Si)-B system and their mechanical properties under compressive loads at room temperature. The alloys of interest were taken from the V-B and V-Si-B systems. Binary V-B alloys were used to review the eutectic composition and the V_3_B_2_ formation depending on the boron concentration. Starting from this point, the eutectic trough was followed into the ternary V-Si-B system by the addition of silicon. These aspects were systematically investigated for the first time, considering the microstructure development and the resulting mechanical properties at room temperature. The results were compared with the current literature in order to estimate the contributions of the various phases to the resulting compressive strengths and to re-evaluate the information available on solidification processes in the V-Si-B system.

Based on the presented experimental results, the following major conclusions can be drawn.

1. The binary V-B alloys show great ductility up to 25% total compressive strain in room temperature compression tests.
2. In contrast to silicon [21], boron seems to have almost no effect on solid solution strengthening which can be attributed to its negligible solubility in the V_SS_ phase. The contribution of Si dissolution can be estimated to be 300 MPa for 1 at.% Si and approximately 600 MPa for 5 at.% Si. It cannot be excluded that oxygen contamination in the V_SS_ phase could have affected the alloys response.
3. The V_SS_ phase controls the deformability in the V_SS_-V_3_B_2_ eutectic microstructure of the V(-Si)-B alloys, whereas the intermetallic V_3_B_2_ phase acts as second phase strengthener.
4. By comparing the results within this work with compression strength data from the current literature, the contribution of V_3_B_2_ second phase hardening in near-eutectic V_SS_-V_3_B_2_ alloys can be estimated to be 400 MPa.
5. With increasing silicon additions from 1 to 5 at.%, the eutectic composition is shifted to lower boron concentrations. Compared to the calculated liquidus projection of the V-Si-B system [18], the present experimental results also suggest a much larger binary eutectic trough than reported recently by da Silva et al. [18]. Thus, more experimental work is recommended to clarify this question.

## Figures and Tables

**Table 1:** Chemical composition, primary phase and phase volume fractions of as-cast V(-Si)-B alloys.

No.	Nominal Composition	Si [at.%]	B [at.%]	Oxygen [wt.ppm]	Primary Phase	V_SS_ [vol.%]	V_3_B_2_ [vol.%]	V_5_SiB_2_ [vol.%]
1	V-0.3B	-	0.3	700 ± 50	V_SS_	97.5	2.5	-
2	V-5B	-	5.2	700 ± 170	V_SS_	87.0	13.0	-
3	V-11.5B	-	11.5	1420 ± 300	V_3_B_2_	78.5	21.5	-
4	V-1Si-10B	1.1	9.9	1480 ± 610	V_3_B_2_	80.0	20.0	-
5	V-1Si-17B	1.0	17.3	930 ± 60	V_3_B_2_	58.5	41.5	-
6	V-5Si-9B	5.2	8.3	1080 ± 250	V_3_B_2_	78.0	14.0	8.0
